# Speculation vs. evidence in the association between e-cigarette use and COVID-19: A response to Soule et al

**DOI:** 10.1016/j.pmedr.2020.101295

**Published:** 2021-08-11

**Authors:** Konstantinos Farsalinos, Raymond Niaura

**Affiliations:** a School of Public Health, University of West Attica, L. Alexandras 196, Athens 11521, Greece; b Laboratory of Molecular Biology and Immunology, Department of Pharmacy, University of Patras, Rio 26500, Greece; c Departments of Social and Behavioral Science and Epidemiology, College of Global Public Health, New York University, New York City, USA

With this letter, we express our concern that the suggestions by Soule et al. (2020) about electronic cigarettes (e-cigarettes) possibly increasing COVID-19 related morbidity and mortality are based on irrelevant and erroneously cited evidence. Additionally, they inappropriately mispresented a blog comment by the first author of this letter.

The authors presented six case reports of “life-threatening lipoid pneumonia” suspected to be associated with e-cigarette use. However, none of them was life-threatening, and lipoid pneumonia is a treatable and reversible condition. Additionally, two of these reports were cases of acute eosinophilic pneumonitis. In two lipoid pneumonia cases, glycerol was implicated as the culprit (McCauley et al., 2012, Viswam et al., 2018). However, this is biologically implausible because glycerol is a polyol, not a lipid. These case reports are irrelevant to COVID-19 and are unlikely to represent general harm, considering that there are millions of e-cigarette users, including long-term users, globally.

The only COVID-19 related study that the authors cited was a cross-sectional survey by Gaiha et al. (2020), which however has some serious flaws. When extrapolating the findings to the general population, Gaiha et al. suggested that, until May 14, 2020, 64.0% of all COVID-19 diagnostic tests in the U.S. were performed in people aged 13–24 years, while 46.8% of confirmed COVID-19 cases were in this age group (Farsalinos and Niaura, 2020). Considering the limited availability of diagnostic resources and the priority given to elderly and people with comorbidities, these findings are implausible and probably represent serious response bias.

Finally, Soule et al. characterized the statement “There is no evidence on any effects of e-cigarettes on coronavirus infectivity and disease progression, and we cannot exclude the possibility that the use of propylene glycol might have some beneficial effects”, written in a blog comment of the first author of this letter (Farsalinos, 2020), as erroneous. Not only is there nothing erroneous in this statement, but Soule et al. grossly mis-presented the blog post. The post cites evidence from studies in the 1940’s, but not the 1980 U.S. Environmental Protection Agency document, as Soule et al. claim. The latter is in fact irrelevant to propylene glycol inhalation through e-cigarettes since it refers to air sanitizers (disinfecting room air), and does not reject the virostatic properties of propylene glycol shown in previous studies. More importantly, the post emphasizes that “these studies do not suggest any effect of propylene glycol on the particular coronavirus strain… Thus, we have no evidence on how e-cigarettes and propylene glycol use may affect disease spread and severity”. Additionally, the post clarifies that there is no evidence that initiating e-cigarette use will prevent SARS-CoV-2 infection or reduce disease severity and, thus, it is not recommended to initiate e-cigarette use. Soule et al. have clearly distorted the overall content of the blog comment by selectively presenting one statement, which is not even erroneous.

In conclusion, Soule et al. speculated on the adverse effects of e-cigarettes on COVID-19 while no such clinical evidence exists until now. Even for smoking, evidence suggests higher odds for adverse outcome among hospitalized patients (Farsalinos et al., 2020a) but lower odds for COVID-19 diagnosis and hospitalization (Hippisley-Cox et al., 2020, Kowall et al., 2020). This has led to the hypothesis that nicotine may have protective properties (Farsalinos et al., 2020a, Farsalinos et al., 2020b). While awaiting the results of ongoing clinical trials of nicotine replacement therapies in COVID-19, it is imperative to refrain from presenting currently-unsubstantiated causal associations and avoid distorting opinions, and it is important to consider that such speculation could discourage smokers from switching to potentially lower-risk e-cigarettes or cause former-smoking e-cigarette users to relapse to smoking.

## Funding

No funding was provided for this letter.

## Declaration of Competing Interest

The authors declare that, for the past 5 years, they have no known competing financial interests or personal relationships that could have appeared to influence the work reported in this paper.
